# Effects of tourism resource search on folk sports project development fuzzy front-end performance: The moderating role of environmental dynamics change

**DOI:** 10.1371/journal.pone.0304161

**Published:** 2024-05-29

**Authors:** Chuanwen Yu, Mengmeng Liu, Hong Yang, Bei Wu

**Affiliations:** 1 School of Physical Education and Health, Heze University, Heze, Shandong, China; 2 General Graduate School, Dongshin University, Naju, Korea; 3 Department of Mathematics, Xinzhou Teachers University, Xinzhou, Shanxi, China; Istinye University: Istinye Universitesi, TURKEY

## Abstract

How to improve the success of new project development through the collection of resources in the preliminary stages of new project development is a new area of research. Given the speed and magnitude of changes in the folk sports market environment, this study analyses the impact of tapping folk tourism resources on sports projects at the project development stage. Through stratified regression analyses of 600 Chinese firms engaged in folk sports project development, this paper finds that tapping internal tourism resources positively affects the Fuzzy Front-End Performance of incremental innovative project development. In contrast, tapping external tourism resources positively affects the Fuzzy Front-End Performance of breakthrough innovation projects. The study also indicates that the speed of environmental change (SEC) positively moderates the relationship between exploring external tourism resources (ERS) and Fuzzy Front-End Performance of incremental innovation projects. In contrast, the magnitude of environmental change (MEC) negatively moderated the relationship between internal tourism resource exploration (IRS) and the FFE-P of similar projects.

## Introduction

With the development of the social economy and the significant improvement of people’s living standards, public demand for tourism and leisure activities has become increasingly diversified and personalized [1]. Against this background, sports tourism projects that combine leisure and recreation with health and sports are increasingly becoming the focus of people’s attention [2]. As a dynamic and potentially emerging industry, sports tourism has gradually been incorporated into the country’s development strategy and has been vigorously promoted and supported by the government. In particular, folk sports tourism, with its rich and unique ethnic culture and sports resources, has rapidly become a highlight in sports tourism [3]. This provides a new direction to promote the diversified development of the sports tourism industry, and it has become an essential element that governments at all levels and relevant departments actively support and promote. China attaches great importance to the development of folk sports tourism, and a series of policies have been introduced, injecting new vitality into the field (Table 1). In March 2010, the State Council issued the "Guiding Opinions on Accelerating the Development of the Sports Industry," which explicitly put forward the integration and development of the sports industry with culture, tourism, and other related fields as a strategic goal, aiming at facilitating the flourishing development of sports tourism and related industries, thus making sports tourism quickly become a new highlight in the development of the sports industry. By December 2016, the State General Administration of Sports and the National Tourism Administration jointly issued the Guiding Opinions on Vigorously Developing Sports Tourism, which further emphasized the strategies of market-led, government support, primary body cultivation, and feature enhancement, focusing on promoting the development of sports tourism activities with unique regional and ethnic characteristics. The successive promulgation of these policies has not only created a superior policy atmosphere for the development of folk sports tourism in China but also greatly stimulated the enthusiasm of all regions in the country to develop folk sports tourism resources and opened up new paths for the promotion of the deep integration of culture and tourism.

**Table 1:** National sports tourism and folk sports tourism-related policy statistics.

Time	Issuing authority	Name of file
March 19, 2010	General Office of the State Council of the People’s Republic of China	Guiding opinions of The General Office of the State Council on accelerating the development of the sports Industry
October 20, 2014	General Office of the State Council of the People’s Republic of China	Several opinions on accelerating the development of sports industry and promoting sports consumption
July 13, 2016	General Administration of sport of China	The 13th Five-Year Plan for the development of sports industry
October 25, 2016	General Office of the State Council of the People’s Republic of China	Guiding opinions on accelerating the development of fitness and leisure industry
	General Office of the State Council of the People’s Republic of China	Outline of the Healthy China 2030 Plan
December 7, 2016	General Office of the State Council of the People’s Republic of China	The 13th Five-Year Plan for tourism development
December 11, 2016	China National Tourism Administration	Guiding opinions on vigorously developing sports tourism
March 9, 2018	General Office of the State Council of the People’s Republic of China	Guiding opinions on promoting the development of all-region tourism
September 4, 2019	General Office of the State Council of the People’s Republic of China	Opinions on promoting national fitness and sports consumption to promote high-quality development of the sports industry

For emerging businesses with relatively limited resources and capabilities, adopting a tourism resource search has become an important strategy to obtain the resources they need to enhance their effectiveness in developing folk sports programs [4]. Investigating and assimilating tourism resources is pivotal for the progression of folk sports projects, enabling businesses to maximize the use of these resources, develop novel folk sports tourism offerings, and consequently propel tourism development forward [5]. Therefore, devising approaches to enhance the quality development of varied folk sports tourism initiatives through effective utilization of tourism resources is essential, aiming at their rejuvenation and presenting a critical challenge in need of prompt attention. Tourism resources encompass a broad range of natural and societal elements capable of attracting visitors, supporting tourism development, and producing economic, social, and ecological advantages.

Tourism resource search refers to the Fuzzy Front-End (FFE) stage of sports enterprises’ development of folk sports projects, in which they conduct sports market research and tourism resource assessment to find suitable tourism resources for development [6]. Tourism resource search by folk sports enterprises can predict the market demand for sports tourism and the feasibility of projects in advance [7]. Through tourism resource search, sports enterprises can better understand the demand of the folk sports tourism market, tourists’ interests, and the feasibility of tourism resources, providing essential predictors and primary data for the development and implementation of sports field [8]. When sports firms have access to a large number of tourism resources during the FFE stage of folk sports field, they can enhance the feasibility, level of innovation, and rate of progress of the folk sports project, thus contributing to the successful implementation of the folk sports project [9, 10]. Existing research has focused on studies of folk sports cultural heritage [11], local dialects [12], and traditional rural villages [13], and few scholars have explored the significance of external tourism assets like colleges, research centers, and travelers in impacting the FFE of folk sports program advancement remains ambiguous.

Knowing the changes in the sports tourism market environment in advance can help sports tourism companies seize tourism market opportunities and reduce investment risks [14]. The development of folk sports projects occurs in a constantly changing external environment, and folk sports firms often adapt their development strategies and decisions to meet market demands and enhance their competitive advantage [15, 16]. At any time during the development of folk sports programs, they are affected by changes in the external environment. Folk sports enterprises need to actively adapt to changes in the external environment to prevent stagnation in the project development process and ensure the successful completion of project development. Thus, the effective search for tourism resources will directly affect the development process of folk sports projects [17–19]. The speed and magnitude of environmental change also affect the complexity of the folk sports project development process to a certain extent [20]. In the current field of research, most of the said research focuses on the search for resources during the progress period of the core project development, and there are few studies on whether the search for tourism resources affects the whole process of folk and sports project development during the ambiguous stage of the development of a new project [21]. Therefore, this study fills this research gap by focus on whether the speed and magnitude of environmental change impact folk sports project development. The main contribution of this study is to demonstrate further the effects of tourism resources on the development of folk sports programs, as compared to previous research [22, 23]. Secondly, the study should focus on the FFE-P of project development. The speed and magnitude of environmental changes determine whether ecological dynamics play a moderating role.

The structure of this paper is organized as follows: It begins with a review of literature on tourism resource exploration, changes in environmental dynamics, and the development of the FFE-P in folk sports projects to formulate research hypotheses and a theoretical framework. This is followed by a detailed methodology section, which covers the sample, data collection methods, measurement instruments, and analysis. The next section validates the research model, tests hypotheses, and discusses the findings. The paper ends with an examination of theoretical and practical consequences, constraints, and prospects for future investigations and findings.

## Literature review

### Tourism resource and folk sports project development

Tourism resources are categorized into internal and external tourism resources [24]. Internal tourism resources involve exploring the inherent assets of the tourism environment, including mountains, lakes, and places of interest, as well as exploring cultural traditions, folk, and customs [25]. Folk sports enterprises can transform these external tourism resources into attractive products [26]. On the other hand, external tourism resources usually result from collaborations with various organizations of interest. These include project development clients, research organizations, and universities [27]. Partnerships between these organizations can be very useful in helping sport tourism enterprises and project development teams to access tourism market information, technological innovations for project development, and sports industry support [28, 29].

Ferreras-Méndez [30] and colleagues identified that ambiguous concepts or vague ideas could lead to increased costs and failures in later stages of product development. Thus, the initial phase of project development, known as the FFE phase, plays a crucial role in the project’s success and is heavily influenced by its level of innovation. It is observed that most folk sports projects follow an incremental innovation strategy, focusing on enhancements and expansions rooted in existing cultural and natural resources [31]. This approach facilitates steady advancement through the continuous refinement and broadening of the project scope. However, a minority of scholars advocate for a more radical approach, emphasizing the need for innovation and disruption in folk sports projects. They suggest introducing groundbreaking ideas and technologies to transcend conventional boundaries, thereby forging unique folk sports experiences [32]. This perspective leads to the classification of folk sports projects into two types: incremental and breakthrough innovation projects. Incremental projects focus on refining and expanding upon current technologies or market demands through adaptive learning, aiming to improve and extend existing knowledge. In contrast, breakthrough projects pursue novel knowledge and technology through exploratory education, challenging and transforming existing technological paradigms to meet new market demands and customer preferences [33].

### Relationships between resource search and sports project

Folk sports company usually actively seek tourism resources to advance the development of folk sports projects and continuously improve the quality of the products of Folk sports projects [34]. By exploring internal tourism resources related to sports, one can attain a thorough comprehension of the available resources within projects, furnishing crucial insights for Folk sports initiatives [35]. In the progressive evolution of innovative project development, the ongoing enhancement of tourism resources is indispensable for achieving project success [36]. Through internal resource searches, companies can not only delve deeper into their distinctive folk sports tourism resources to bolster project distinctiveness and innovation but also anticipate potential risks in advance, offering timely feedback and guidance to tackle challenges [37]. Internal resource searches provide critical information for the initial planning and of folk sport project innovative project development [38]. As Folk sports projects continue to be developed, they may need more internal tourism resources or search for ineffective tourism resources. This directly leads to the stagnation of innovative folk sports projects or a bottleneck phase in developing folk sports projects [39]. Therefore, folk sports enterprises need to optimize and innovate internal tourism resources to increase the demand for new project development [40]. These techniques can solve the problem of internal tourism resource constraints in Folk sports enterprises [41]. The following hypothesis is therefore proposed:

H1a: IRS positively impacts the FFE of incremental innovation project.
H1b: IRS exhibits a curvilinear relationship, characterized by an inverted U-shaped effect on the FFE of breakthrough innovation project development.

External resource searches can help folk sports businesses discover several resources external to the starting sports market, which provide vital information for the initial planning of folk sports projects and can help to facilitate the development of folk sports projects. However, excessive external tourism resources during the development of folk sport projects may lead to fragmentation of tourism resources, redundancy of information, and complication of leadership decisions. Over-reliance on external tourism resources and thus neglecting the optimization of internal tourism resources in folk sports enterprises can hinder the progress of folk sports projects [42]. Therefore, when the influx of external tourism resources in Greater Lyon is increasing, folk sports enterprises need to reduce the need for external resources through humidity and fully integrate internal tourism resources to promote the development of folk sports programs [43]. When folk sports enterprises face breakthrough folk sports project development, external tourism resources can encourage folk sports enterprises to proactively explore new tourism resources and development opportunities [44]. By developing potential tourism resources, folk sports enterprises continue exploring untapped external resources to support folk sports projects [45]. Through an in-depth exploration of external resources, folk sports enterprises can precisely delineate the problems in the competitive sports market, providing folk sports enterprises with crucial information for project definition [46]. In addition, external tourism resources can identify innovative teams in time to collaborate with other tourism operators associated with the industry, obtaining novel first-hand resources and expertise to stimulate the overall development of innovative folk sports projects [47]. External tourism resources provide folk sports businesses with greater flexibility in terms of sports market dynamics and the needs of tourism customers, enabling them to better adapt to changes in the sports market environment. The following hypothesis is therefore proposed:

H2a: The ERS demonstrates an inverted U-shaped impact on the FFE of incremental innovation project development.
H2b: The ERS positively influences the FFE of breakthrough innovation project development.

### The moderating role of environmental dynamics change

Stieglitz [44] and his team observed that the unpredictable nature of environmental effects primarily stems from the rapid pace and significant scale of ecological changes driven by market and technological demand fluctuations. Past studies have often mixed up the concepts of the speed and magnitude of ecological changes, thereby muddling both the temporal and categorical aspects of environmental dynamics. This confusion has hindered the development of explicit explanations for these phenomena. Environmental Dynamics Change speed is the rate at which environmental conditions or factors evolve. The interplay between the speed and scale of changes in the external environment plays a crucial role in adding to the uncertainty encountered during the FFE-P of project development, influencing decision-making and strategic planning processes [48, 49]. When the speed and magnitude of changes in the external environment change rapidly and significantly, folk sports project development faces more unknowns and uncertainties [50]. In an environment where the sports tourism market and innovative technologies are constantly changing, folk sports companies have ample time to explore strategies related to project development. The phase of breakthrough innovation project development can reshape the structure of the current sports market and generate distinctive and valuable folk sports programs that are challenging to replicate. As a result, when folk sports project development is relatively stable, it can bring considerable profits and sustained revenue to folk sports companies [51]. On the contrary, when folk sports programs are faced with a rapidly changing external environment during the development phase, folk sports companies need to seize opportunities quickly in the sports market. And in this rapidly changing environment, incremental innovation projects with cost advantages show unique advantages. Incremental innovation projects can promptly adapt to the demand of the sports market and reduce the risk and uncertainty in the project development process. These factors can enable folk sports businesses to generate profits quickly and grow steadily [52]. However, the inherent scarcity of folk sports businesses in different environments limits their ability to adapt the flexibility of their innovation strategies to use new environments [53]. This shared knowledge, complex social relationships, and structural embedding can help folk sports firms quickly identify scarce resources when searching for tourism resources [54]. With inertial mechanisms, folk sports firms can leverage past solutions and experiences to mitigate losses and minimize friction costs, thus increasing the efficiency of knowledge transformation and resource integration [55]. We propose the following hypothesis:

**H3a:** SEC positively strengthens the impact of IRS on FFE-P in incremental innovation project development.
**H3b:** SEC dampens the effect of IRS on FFE-P in breakthrough innovation project development.
**H4a:** SEC enhances the impact of ERS on FFE-P in incremental innovation project development.
**H4b:** The SEC dampens the impact of ERS on FFE-P in breakthrough innovation project development.

Chinese folk sports enterprises rely more on internal technological updating and external resource utilization to adapt to the ever-changing sports market environment. Changes in the external resource environment may result in the progress of folk sports programs and hinder the profitability of folk sports enterprises [56]. Folk sports firms that rely on the development of incremental innovation projects may need to be faster to grow. In contrast, firms that choose breakthrough innovation projects will adapt to the changing environment in a short period. Despite the high cost of maintaining the strategic flexibility of folk sports firms, when faced with rapid changes in the external environment, folk sports firms are more inclined to adopt flexibility strategies aligned with environmental changes [57]. When there are inherent resource constraints in folk sports firms, they need to match sports markets and resources to enhance flexibility and organizational coordination in resource allocation. In this mechanism, the search for tourism resources by folk sport firms is critical, and there is a greater demand for resources that enhance the flexibility of folk sport firms in particular. Having high flexibility of tourism resources can provide strong technical support for breakthrough innovative projects at the FFE stage of folk sports project and mitigate the negative impact of lack of resources on folk sports project development [58, 59]. We propose the following hypothesis:

**H5a:** The MEC diminishes the influence of IRS on FFE-P in incremental innovation project development.
**H5b:** The MEC enhances the influence of IRS on FFE-P in breakthrough innovation project development.
**H6a:** The MEC dampens the impact of ERS on FFE-P in incremental innovation project development.
**H6b:** The MEC strengthens the impact of ERS on FFE-P in breakthrough innovation project development.

**Fig 1:**
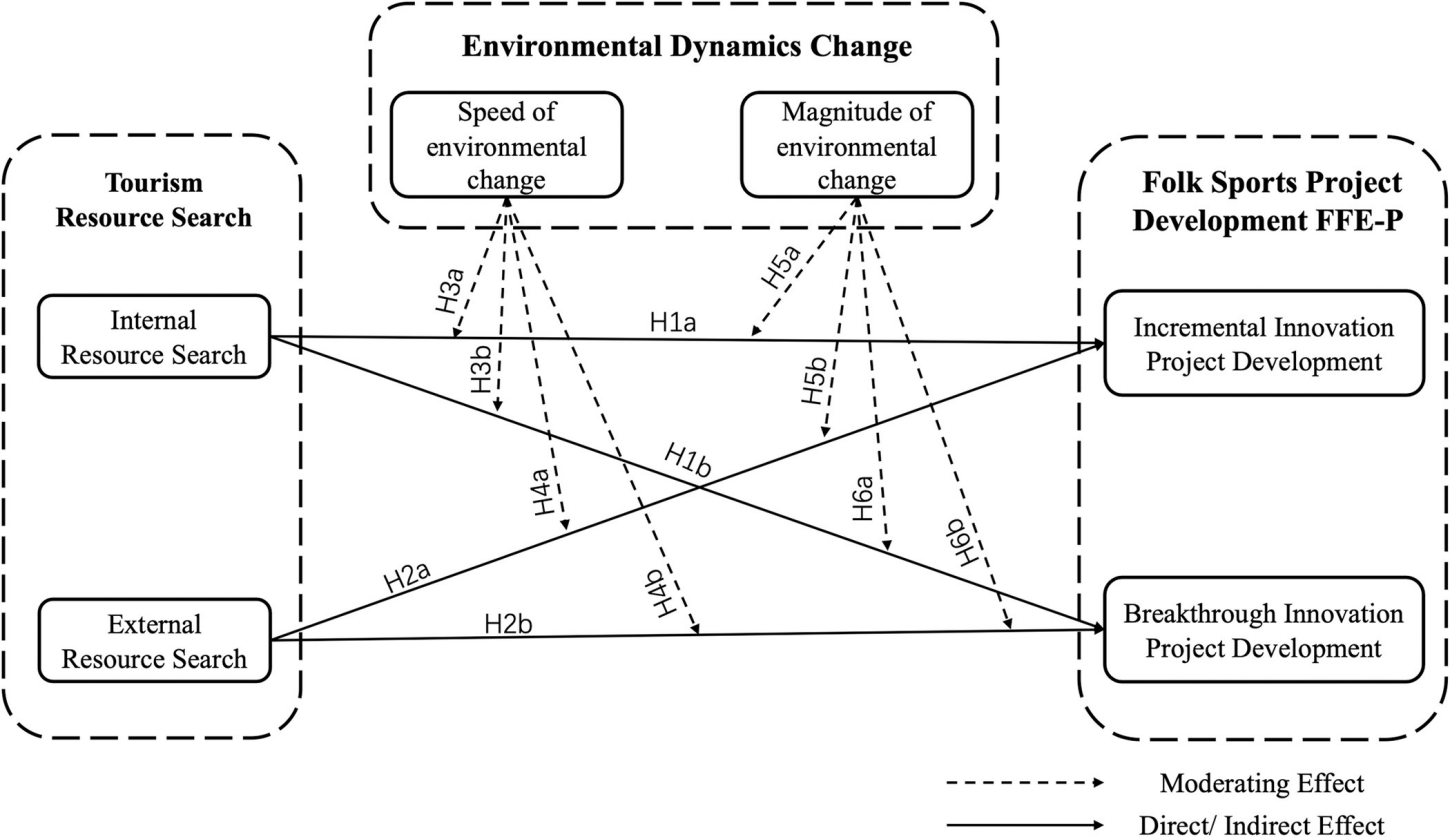
Research model.

The conceptual framework diagram for this study is shown in Fig 1.

## Methodology

### Sample and data collection

For our study’s empirical investigation, we opted for a survey method based on questionnaires. This method is advantageous due to its ease of replication, capability to evaluate multiple variables simultaneously, and potential to enhance the generalizability of findings. The data collection process unfolded in three sequential phases. Initially, we crafted a survey tool informed by existing research and tailored it to the specific context of China with insights from scholars and industry veterans. A preliminary test with a random selection of 8 firms helped refine the survey’s clarity and brevity. Next, we refined our sample selection with recommendations from educational and governmental bodies, undertaking pre-survey phone calls to improve response rates. Participants were promised a summary of the findings to encourage participation. The final phase of survey distribution occurred between October 1, 2022 to March 14, 2023, targeting a randomized group of 1000 Chinese companies specializing in folk sports. The survey was emailed to their senior managers, detailing the study’s purpose and ensuring confidentiality. Follow-up reminders were sent via email to maximize responses. Out of the distributed surveys, 600 were returned completed, obtaining a 60% response rate. The demographic characteristics of the sample are outlined in Table 2.

**Table 2:** Demographic profile of the participants.

Company attributes	Frequency	Percentage(%)
Gender		
Male	323	53.8
Female	277	46.2
Company scale		
<50	364	60.7
50–100	160	26.7
>100	76	12.7
Age of business		
<5 years	306	51.0
5–10 years	224	37.3
>10 years	70	11.7
Type of position		
Administrators	200	33.3
Project developers	400	66.7

As discussed by Wong et al. [60], our study acknowledges that there may be a risk of standard method bias (CMB) due to data collected from a single source at a single point in time, which may threaten the validity of our findings. We employ an integrated strategy in our study to address and assess the extent of CMB. First, we use Harman’s one-factor test, a widely recognized technique for determining the presence or absence of CMB [61]. This analysis yielded six different factors with eigenvalues greater than or equal to 1.0, accounting for 72.8 percent of the total variance, of which the principal factor accounted for 22.3 percent. Variance dispersion across multiple factors indicates dilution of the standard method variance. Subsequently, we conducted confirmatory factor analysis (CFA) within the framework of Harman’s one-factor test to investigate the data further. The CFA results displayed by various fit indices, CMIN/DF = 1.886, CFI = 0.973, TLI = 0.956, RFI = 0.974, and RMSEA = 0.034, suggest the presence of several different factors rather than a dominant common method factor. To further mitigate Common Method Bias (CMB), we enhance the measurement model by incorporating a method factor into the Confirmatory Factor Analysis (CFA) model. This adjustment led to a marginal enhancement in the fit index of the model. The results show that the method factor contributes very little to the overall variance, providing further evidence that the CMB has a minimal impact on our study. To mitigate the potential effects of CMB in advance, the questionnaire was carefully arranged to separate the items related to the independent, mediating, and dependent variables in different sections. This structural separation aims to minimize the bias generated during survey design and implementation, ensuring our research results are more reliable and valid.

### Variables and measures

In this study, tourism resources are categorized into internal and external tourism resources. Internal tourism resources include some localized elements of the folk sports enterprise, such as local characteristics, intangible cultural heritage, and historical sites of the location of the folk sports enterprise [62], while external tourism resources include resources from scientific research institutes, universities, and cooperating customer groups in the location of the sports enterprise. The internal tourism resource search is more concentrated on developing folk sports projects [63]. The external tourism resource search is a more extensive search method due to its more comprehensive scope. Although still centered on tourism resources, this search method uses external sources of knowledge and provides resources other than internal information for folk sports project development.

Averina [64] and colleagues outlined the critical differences between incremental and breakthrough project development, focusing on the initial FFE stage. Projects are classified into incremental or breakthrough types, depending on the level of innovation they introduce. The performance of these projects during the FFE stage, known as FFE-P, accurately gauges the development success of folk projects. From a features viewpoint, we advocate for strategies aimed at diminishing uncertainty and clarity while encouraging the reinforcement and continual development of products in the FFE stage. Looking at outcomes, the products resulting from the FFE phase serve as a vital base. Therefore, we highlight the importance of the product’s initial concept, detailed definition, and early planning stages as critical indicators for evaluating the execution quality of activities within the FFE phase.

The rate of Environmental Dynamics Change (EDC) is defined by how quickly environmental factors or conditions change over time, while the magnitude of EDC is measured by the level of variation from their previous states. In examining ecological dynamics, we adopt the segmentation approach outlined by Rexhäuser and Rammer [65] focusing on a binary model that distinguishes between the speed and scale of environmental changes.

To improve the accuracy and reliability of our research findings, we introduce company size as a primary control variable, measured by the natural logarithm of the number of employees. The year of the company’s foundation is also considered a control variable to improve the research results’ reliability further.

### Reliability and validity

In this study, the reliability and validity of the questionnaire were analyzed through exploratory factor analysis. The application of the varimax rotation method carried out a preliminary assessment of individual dimensions. Six expected constructs were isolated through the rotated load factors, which together accounted for 72.8 per cent of the variance. This indicates that the respective themes of our measurement scales fit well with the subjects and the constructs measured. By validating Cronbach’s alpha and composite reliability (CR), the test results indicated that the Cronbach’s alpha and CR scores for each construct exceeded the established threshold of 0.80, highlighting their high reliability, as detailed in Table 3.

**Table 3:** Reliability testing of concepts.

Variable	Indicator	Factor Loading	AVE	CR	Cronbach’s α
Internal Resource Search	IRS1	0.82	0.67	0.89	0.85
	IRS2	0.85			
	IRS3	0.85			
	IRS4	0.76			
External Resource Search	ERS1	0.85	0.71	0.91	0.88
	ERS2	0.84			
	ERS3	0.87			
	ERS4	0.83			
Incremental Innovation Project FFE-P	IIP1	0.86	0.73	0.93	0.92
	IIP2	0.87			
	IIP3	0.86			
	IIP4	0.87			
	IIP5	0.82			
Breakthrough Innovation Project FFE-P	BIP1	0.73	0.60	0.88	0.88
	BIP2	0.71			
	BIP3	0.80			
	BIP4	0.82			
	BIP5	0.81			
Speed of Environmental Change	SEC1	0.76	0.57	0.84	0.86
	SEC2	0.78			
	SEC3	0.76			
	SEC4	0.72			
Magnitude of Environmental Change	MEC1	0.94	0.79	0.94	0.92
	MEC2	0.94			
	MEC3	0.85			
	MEC4	0.81			

See S1 Appendix

In this study, the validity of the model was assessed through a validated factor analysis (CFA). Measurement scales for each variable were first developed based on existing literature. The validation results of CFA showed that CMIN/DF = 1.889<3; TLI = 0.954>0.9; CFI = 0.960>0.9; and RMSEA = 0.036<0.05. These data suggest that this study’s model fit was all within the acceptable range. In addition, we also provide Average Variance Extracted (AVE) values above 0.8, indicating that the convergent validity of the data in this study is within a reasonable range. The AVE for each construct exceeded the benchmark of 0.50, as shown in Table 4. Furthermore, the correlation coefficients between constructs were all below the threshold of 0.9, indicating that multicollinearity did not adversely affect our results. An additional investigation into multicollinearity by calculating the Variance Inflation Factor (VIF) revealed that no VIF value surpassed the critical threshold of 10, confirming the absence of multicollinearity concerns in our research.

**Table 4:** Inter-conceptual correlation analysis.

Variable	Mean	SD	IRS	ERS	PIP	BIP	SEC	MEC
IRS	4.46	0.53	0.84					
ERS	3.45	0.41	0.12*	0.93				
IIP	4.56	0.52	0.32**	0.27**	0.82			
BIP	3.38	0.54	0.45	0.37**	0.29*	0.73		
SEC	3.23	0.46	0.11	0.13**	0.14*	0.58**	0.78	
MEC	4.79	0.67	0.25**	0.12**	0.24**	0.36	0.26	0.87

See S1 Appendix

## Results

In our research, Model M1 integrates both control and independent variables to examine their effects on the FFE-P of projects focusing on incremental innovation. A Durbin-Watson test, yielding a value of 1.907, alongside regression tolerance figures surpassing 0.1, confirmed the independence of residuals, verifying the model’s validity. This model revealed that both IRS and ERS exert an affirmative, significant influence on FFE-P, with IRS (β = 0.238, p<0.001) and ERS (β = 0.386, p<0.001) demonstrating significant predictive capability for FFE-P outcomes, thereby affirming Hypothesis 1a.

Model M2 examines the role of moderating variables and introduces the squared term of ERS, investigating its potential nonlinear effect on FFE-P. The findings indicated no significant curvilinear relationship (β = 0.001, p>0.05), leading to the non-support of Hypothesis 2a. However, the model identified a substantial positive impact of Magnitude of Environmental Change (MEC) on FFE-P (β = 0.389, p<0.001), whereas Speed of Environmental Change (SEC) did not show a significant effect, challenging some of the hypothesized relationships.

In Model M3, the interaction between SEC and the squared term of ERS, alongside IRS and MEC, was analyzed, revealing a significant favorable influence of the former (β = 0.043, p<0.05) and a significant negative effect of the latter (β = -0.427, p<0.01) on FFE-P, supporting hypotheses H4a and H5a, respectively. Other interactions, such as IRS with SEC and the squared term of ERS with MEC, did not exhibit significant impacts, leading to the rejection of Hypotheses H3a and H6a. Model M4 assessed the FFE-P for projects focusing on breakthrough innovation, noting a significant positive effect of ERS (β = 0.476, p<0.001), thereby supporting Hypothesis 2b, while IRS did not show a significant impact. Model M5 included:

- Moderating variables and the squared term of IRS.
- The latter was found to have no significant effect on FFE-P.
- Leading to the rejection of Hypothesis 1b.

The analysis also highlighted a significant positive effect of the magnitude of environmental change, though the speed of environmental change did not significantly impact FFE-P for breakthrough innovation projects.

Lastly, model M6 investigated the moderating impacts of the pace of environmental change on the association between tourism resource exploration and FFE-P, finding no significant interaction effects, thus leading to the rejection of the relevant hypotheses. This comprehensive analysis, detailed further in Table 5., underscores the nuanced influences of resource searches and environmental dynamics on the innovation project development’s early stages.

**Table 5:** Results of the segmented regression analysis.

Variables	Incremental Innovation Project FFE-P			Breakthrough Innovation Project FFE-P		
	M1	M2	M3	M4	M5	M6
Control variables						
Employee count	0.024	0.018	0.028	-0.003	-0.055	-0.068
	(0.413)	(0.347)	(0.553)	(-0.042)	(-1.182)	(-1.453)
Age of business	0.063	0.048	0.071	-0.037	-0.013	-0.004
	(0.971)	(0.813)	(1.279)	(-0.571)	(-0.282)	(-0.082)
Independent variables						
IRS	0.238***	0.147*	0.111	0.126	-0.330	-0.196
	(2.947)	(1.953)	(1.521)	(1.336)	(-1.664)	(-0.652)
ERS	0.386***	0.147	-0.618	0.476***	0.071	0.069
	(4.514)	(0.401)	(-0.693)	(5.004)	(0.909)	(0.865)
IRS^2^					0.053	0.030
					(1.902)	(0.673)
ERS^2^		0.001	0.108			
		(0.015)	(0.905)			
Moderating variables						
SEC		0.061	0.068		0.116	0.185*
		(0.741)	(0.753)		(1.396)	(1.935)
MEC		0.389***	0.290**		0.659***	0.637***
		(4.503)	(3.192)		(8.737)	(7.911)
Interactive items						
IRS^2^×SEC						0.031
						(1.572)
ERS×SEC						0.010
						(0.081)
IRS^2^ ×MEC						-0.024
						(-1.262)
ERS×MEC						-0.019
						(-0.156)
IRS×SEC			0.028			
			(0.334)			
ERS^2^×SEC			0.046*			
			(2.478)			
IRS×MEC			-0.427**			
			(-3.132)			
ERS^2^×MEC			-0.037			
			(-0.219)			
R^2^	0.425	0.453	0.623	0.434	0.698	0.707
ΔR^2^	0.397	0.505	0.573	0.396	0.686	0.687
F	21.075***	18.813***	15.887***	29.827***	55.811***	36.144***

Notes: n = 300

***p < 0.001

** p < 0.01

* p < 0.05 (two-tailed test), T-value in brackets. See S1 Appendix

**Fig 2:**
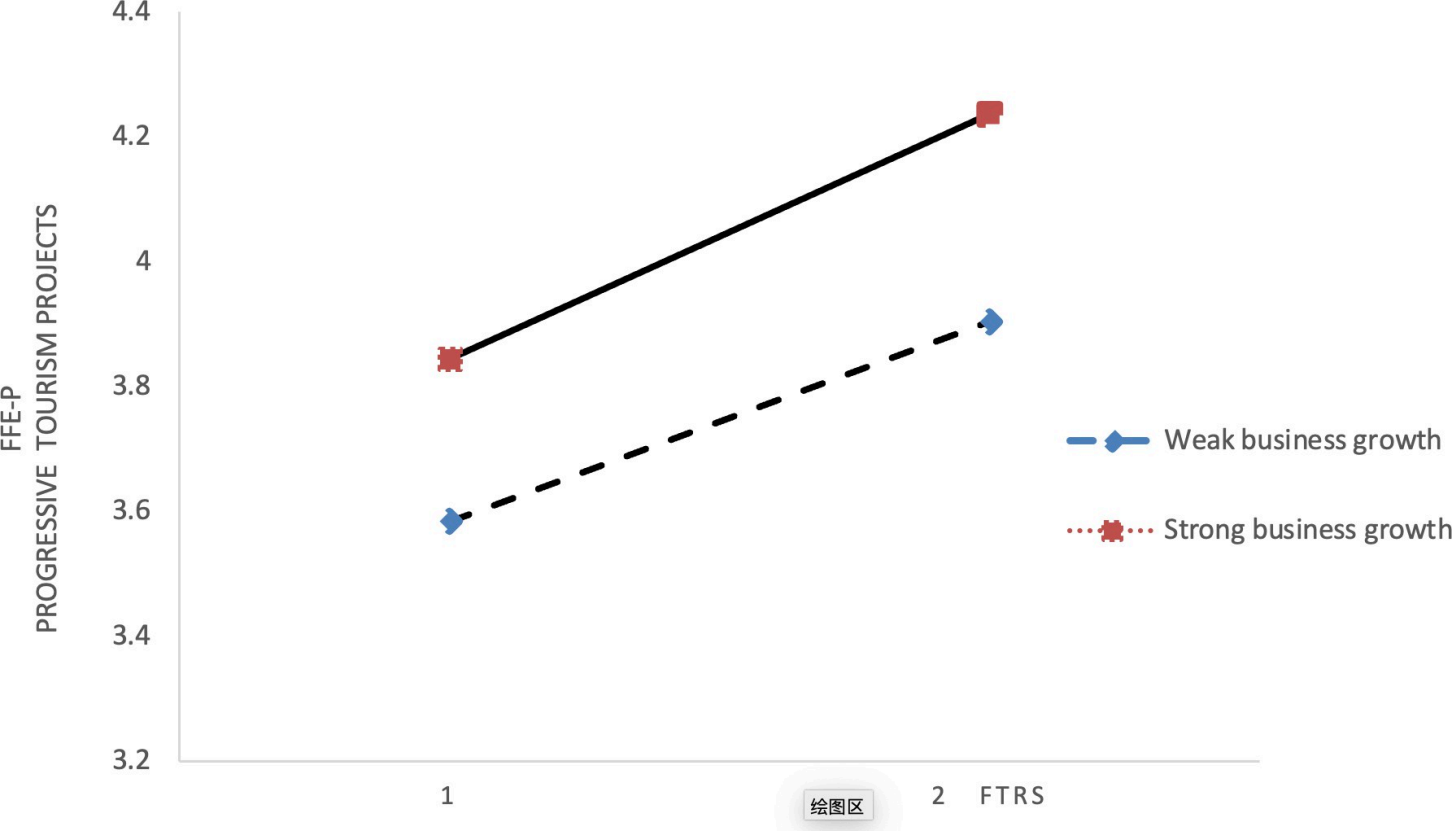
Interplay between SEC and IRS.

**Fig 3:**
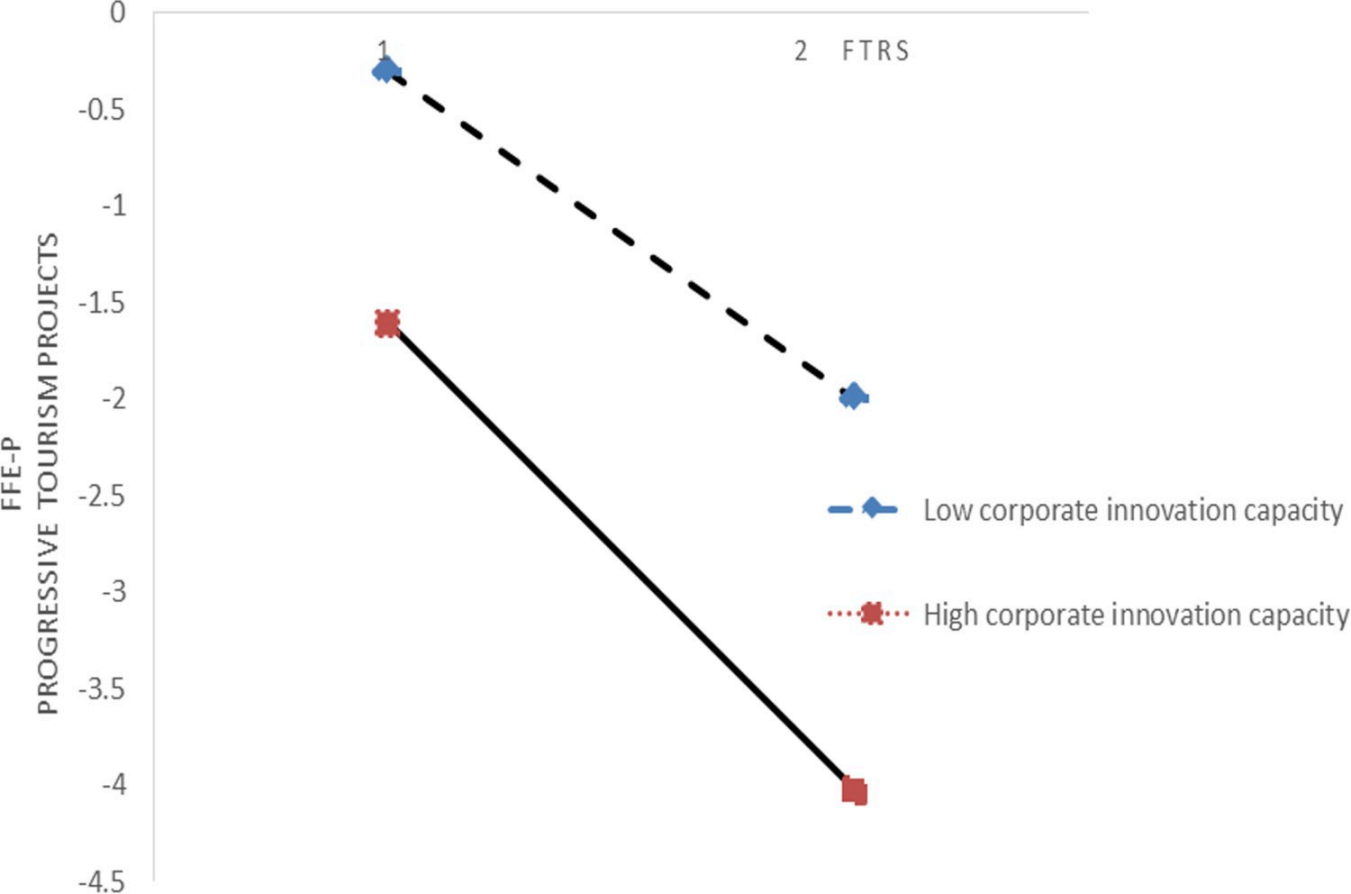
Interplay between MEC and IRS.

This research employs visual aids, specifically interactive relationship graphs, to depict the moderating effects under investigation. Figs 2 and 3 elucidate how changes in environmental dynamics influence the interplay between IRS and FFE-P in projects targeting incremental innovation. These figures highlight that as environmental dynamics change, IRS significantly influences the FFE-P of such projects, with its impact being notably affected., suggesting a nuanced interplay between internal resource exploration and ecological factors in fostering the development of incremental innovation projects.

**Fig 4:**
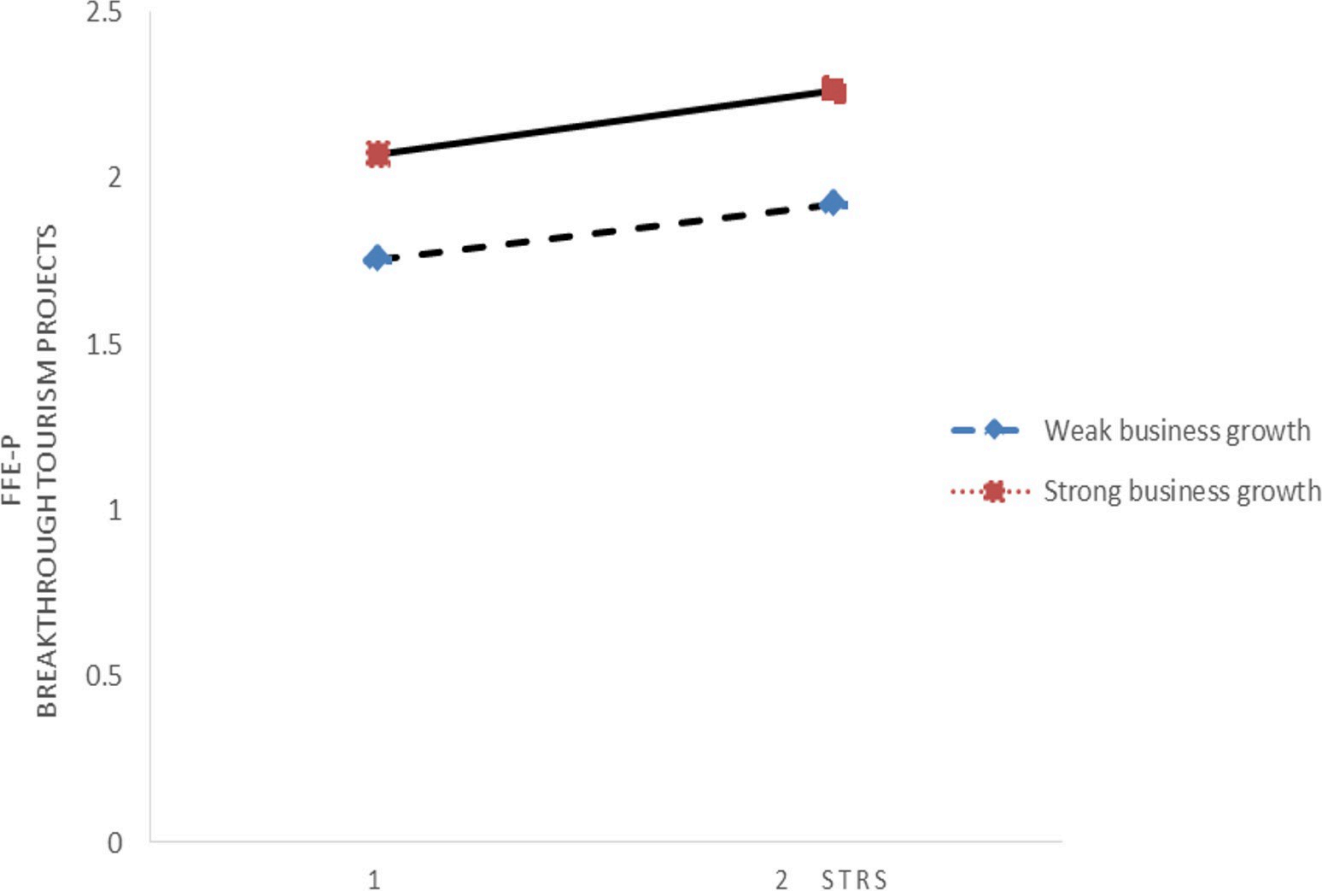
Interplay between SEC and ERS.

**Fig 5:**
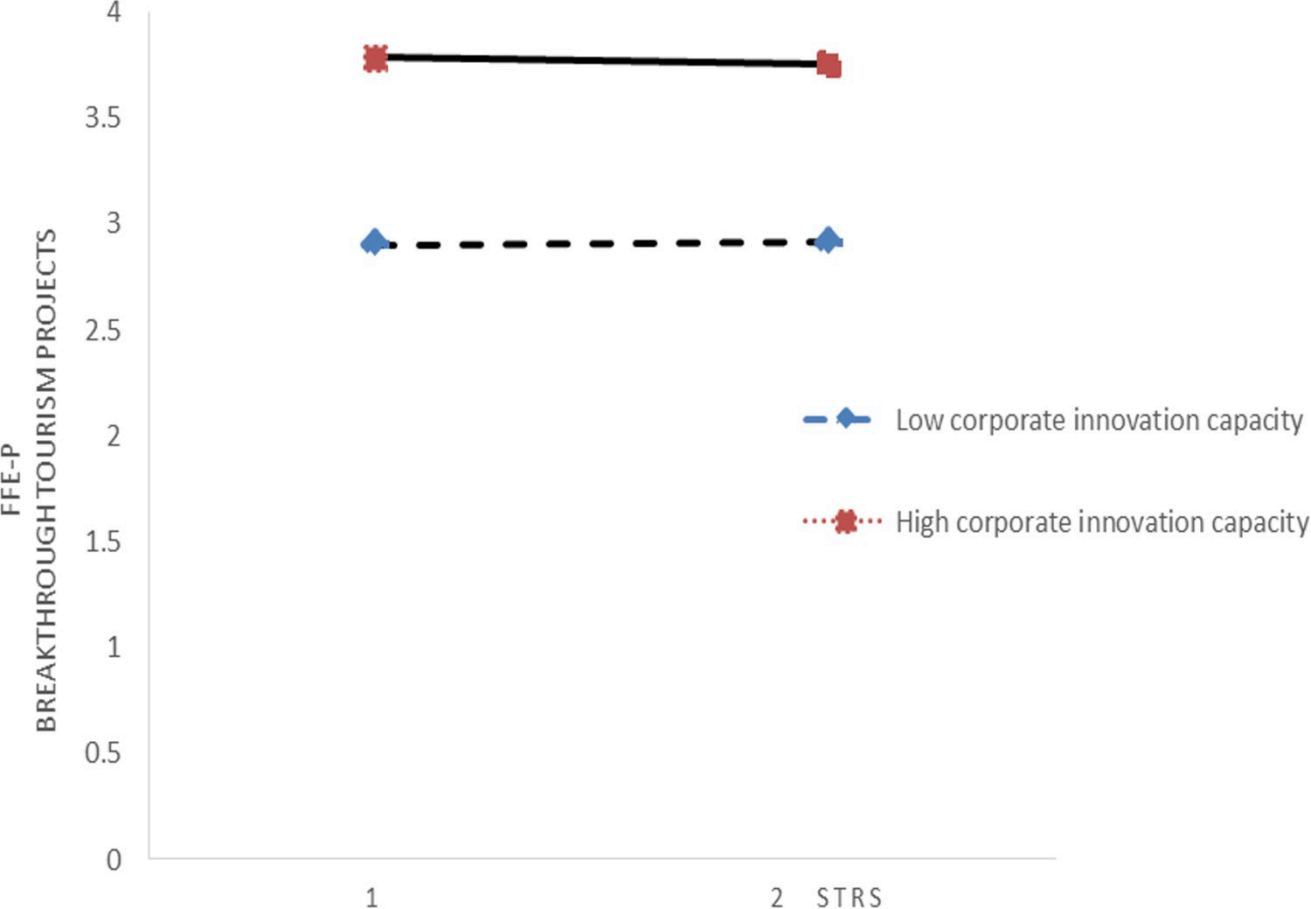
Interplay between MEC and ERS.

Conversely, Figs 4 and 5 focus on the influence of environmental dynamics on the relationship between ERS and the FFE-P in the context of breakthrough innovation project development. These graphs reveal that the moderating effect of environmental dynamics on this relationship is minimal, indicating that changes in the external environment do not significantly alter the impact of ERS on the FFE-P of projects geared towards groundbreaking innovations.

The distinctions drawn from these figures, supported by the underlying table data, suggest a differential impact of environmental dynamics based on the type of innovation (incremental vs. breakthrough) and the source of resource search (internal vs. external). While environmental dynamics play a crucial role in modifying the effectiveness of IRS in incremental innovation projects, their influence is less pronounced when examining the effect of ERS on breakthrough innovation projects.

## Discussion

Folk sports enterprises seek internal tourism resources and use them to enhance the process of developing folk sports programs that are mainly progressive. At the same time, in the same area, folk tourism enterprises that seek external tourism resources rely primarily on developing folk sports programs based on ground-breaking and innovative projects. These internal and external tourism resource searches can positively influence folk sports project enterprises in the ambiguous front-end stage of project development [66, 67]. It can be seen that folk sports enterprises seeking external tourism resources need to introduce new ideas and technologies as a basis, and folk sports enterprises should invest heavily in developing and applying external tourism resources. This search for external resources is more comprehensive than information and resources obtained from various stakeholders, including cooperative customer groups and scientific institutions, which folk sports enterprises utilize in order to facilitate project development and maintenance. At the same time, to maximize the strategic benefits of these resources, folk, and sports enterprises need to assess the objectives and nature of the enterprise and the sports market environment in which the folk and sports enterprise operates. By adapting the approach to tourism resource search, folk, and sports enterprises can better position themselves to achieve the desired outcomes.

Although current research has recognized the importance of tourism resources for folk sports project development, there needs to be a theory provided in previous research to elucidate the moderating role of changing environmental dynamics between resource and folk sports project development. In some cases, changes in ecological dynamics may exacerbate or attenuate this relationship. The results of this study are similar to those of Lütjen et al. [68] in that external resource search is more critical for folk sports firms in the face of changing environments. Our findings show that external resource search has a more significant impact on incremental innovation projects in the context of rapid environmental change [69]. Incorporating external tourism resources into folk sports project development to improve the information processing capacity of folk sports enterprises [70, 71]. Such a foundation would encourage external tourism resources to move toward incremental folk sport innovation projects as firms face rapid environmental change [72].

However, there are still some things that could be improved in the research methodology. Firstly, Harman’s one-factor test and CFA were used in our study to assess common method bias. To assess and mitigate the effects of common method bias, relying on single-source data may still introduce biases that cannot be eliminated. Future studies may consider using multi-source or longitudinal data to enhance the robustness of the study further [73]. Secondly, due to the uniqueness of the folk sports business market, it is more important to incorporate local cultural factors and the influence of the sports market environment when interpreting the results of the study. Therefore, future research can further enhance the reliability and validity of the study by collecting data from multiple sources, adopting a more diverse sample, increasing the breadth and depth of questionnaire pre-testing, and adopting more advanced data analysis techniques [74, 75].

### Implications for theory

This study makes several significant theoretical contributions to existing literature.

Firstly, it deepens the understanding of the importance and complexity of tourism resource searches, highlighting that such searches come with inherent costs and risks. The importance of choosing tourism resources can be effectively adjusted in a given context. The relationship between tourism resources and project development establishes a comprehensive modelling and clinical framework that encompasses the speed and magnitude of environmental change in the sports market. The study also illustrates how folk sports businesses can make the right adjustments in the face of a changing sports market.

Secondly, by studying the speed and magnitude of changes in the folk sports market environment we can learn that folk sports programme managers can make timely policy decisions and adjustments according to changes in the sports market.

Finally, this study analyses the non-linear moderating effect of environmental changes in the folk sports market through the search for tourism resources. It complements the gap in exploring the influence of tourism resources on the FFE-P of folk sports. This offers a valuable framework for future research on inter-organizational dynamics and project development processes within the folk sports and tourism domains.

### Implications for practice

The findings of this study have practical implications for managers involved in the development of folk sports programs.

Flexibility in Resource Allocation: Managers should leverage their organization’s capability for the flexible distribution of tourism resources to meet the nuanced demands of different folk sports projects. For sports enterprises centered on progressive folk sports projects, improving the innovative technical and managerial skills of the development team is crucial. Folk sports enterprises should encourage interaction with academic and research institutions. However, project managers should also be wary of the possible negative effects of too much collaboration, such as information overload and reduced efficiency in absorbing new information.

The ability to innovate and adapt to market changes beyond the inherent: folk sports enterprises should adopt a dynamically changing tourism resource search model to adapt to the development of folk sports projects. Moderately adjust the focus of tourism resource search to avoid the pitfalls of resource scarcity and misalignment of supply and demand in the folk sports market. Folk sports project development managers must maintain a high degree of vigilance on the sports tourism market, monitor changes in the external market at any time, and be prepared to adjust the allocation of tourism resources and optimize the project development strategy accordingly.

Differentiation strategies based on the dynamics of the sports market environment: Management strategies of folk sports programs should evolve with the speed and magnitude of changes in the sports market environment. In rapidly changing environments, folk sports enterprises should promptly adjust project development models and strengthen sports tourism market regulation and other rapid adaptation measures [76]. On the contrary, when the sports market environment is changing rapidly, folk sports enterprises must thoroughly reassess sports tourism resource allocation and project development workflow. Such strategic shifts ensure the project remains competitive and aligned with the new environmental conditions.

We encourage managers of folk sports program development to take a proactive stance and use adaptive strategies to optimize brigade resources to ensure the success of folk sports programs in a diverse and ever-changing sports market environment.

## Conclusion

Our research focuses on analyzing the relationship between tourism resources and the development of sports projects and delves into how to go about tapping into tourism resources that are useful to sports businesses. Through continuously discovering resources, we can improve the success rate of folk sports enterprises in the project development stage and effectively avoid investment risks. In addition, we also discuss the effective use of resources to avoid risks when folk sports enterprises face changing external environments. This kind of research provides a new theoretical perspective for the management and effective development of folk sports enterprises and enhances the understanding of tourism resources by folk sports enterprises. The main results of this study demonstrate that the accumulation of internal tourism resources can effectively promote effective project development in folk sports enterprises that focus on incremental innovation projects. In contrast, folk sports enterprises that rely on external tourism resources depend more on developing breakthrough innovation projects. Ultimately, it was discovered that the velocity and scale of environmental shifts also positively influenced the moderation of the relationship between resource exploration and project development. Conversely, the MEC adversely moderates the impact of IRS on the FFE-P of such projects. Despite its contributions, the study acknowledges certain limitations. It focuses exclusively on the moderating role of environmental dynamics, leaving room for future research to explore the influence of environmental uncertainty and complexity on this relationship. Additionally, while the study illuminates the impact of tourism resource search under the lens of ecological dynamic changes, it recognizes that these are not the sole moderating factors at play. Future studies are encouraged to expand the research model to consider other potential moderators, such as organizational culture, the duration of involvement in projects, and additional performance indicators like innovation costs, delivery, and flexibility. These directions underscore the need for a broader exploration of the factors influencing project success in the context of folk sports and highlight the potential for interdisciplinary approaches to enhance our understanding of how environmental and organizational variables interact to shape project outcomes.

## Supporting information

S1 Appendix (DOCX)
