# Supplementary material for: Effects of tourism resource search on folk sports project development fuzzy front-end performance: The moderating role of environmental dynamics change
Source: PLoS One. 2024 May 29;19(5):e0304161. doi: 10.1371/journal.pone.0304161 (PMC11135685; doi:10.1371/journal.pone.0304161)
Supplement: S1 Appendix — (DOCX) [file pone.0304161.s001.docx]

**Table 6 S1 Appendix**

| Serial number | Full name | Abbreviations |
| --- | --- | --- |
| 1 | Fuzzy Front-End | FFE |
| 2 | Fuzzy Front-End Performance | FFE-P |
| 3 | Speed of Environmental Change | SEC |
| 4 | External Resource Search | ERS |
| 5 | Environmental Dynamics Change | EDC |
| 6 | Incremental Innovation Project | IIP |
| 7 | Breakthrough Innovation Project | BIP |
| 8 | Common Method Bias | CMB |
| 9 | Confirmatory Factor Analysis | CFA |
| 10 | Exploratory Factor Analysis | EFA |
| 11 | Average Variance Extraction | AVE |
| 12 | Variance Inflation Factor | VIF |
| 13 | Internal Resource Search | IRS |
| 14 | External Resource Search | ERS |
| 13 | Magnitude of Environmental Change | MEC |
| 14 | Incremental Innovation Project | IIP |
| 15 | Breakthrough Innovation Project | BIP |
